# Comprehensive Identification of *Krüppel-Like Factor* Family Members Contributing to the Self-Renewal of Mouse Embryonic Stem Cells and Cellular Reprogramming

**DOI:** 10.1371/journal.pone.0150715

**Published:** 2016-03-04

**Authors:** Hyojung Jeon, Tsuyoshi Waku, Takuya Azami, Le Tran Phuc Khoa, Jun Yanagisawa, Satoru Takahashi, Masatsugu Ema

**Affiliations:** 1 Department of Anatomy and Embryology, Faculty of Medicine, University of Tsukuba, 1-1-1 Tennoudai, Tsukuba, Ibaraki, 305–8577, Japan; 2 Graduate School of Pharmaceutical Sciences, The University of Tokyo, Hongo, Bunkyo-ku, Tokyo, 113–0033, Japan; 3 Department of Anatomy and Embryology, Human Biology Program, School of Integrative and Global Majors, University of Tsukuba, 1-1-1 Tennodai, Ibaraki, 305–8575, Japan; 4 Graduate School of Life and Environmental Sciences, University of Tsukuba, 1-1-1 Tennodai, Tsukuba, Ibaraki, 305–8577, Japan; 5 Center for Tsukuba Advanced Research Alliance, University of Tsukuba, 1-1-1 Tennodai, Tsukuba, Ibaraki, 305–8577, Japan; 6 International Institute for Integrative Sleep Medicine, Life Science Center, University of Tsukuba, 1-1-1 Tennodai, Tsukuba, Ibaraki, 305–8577, Japan; 7 Animal Resource Center, University of Tsukuba, 1-1-1 Tennodai, Tsukuba, Ibaraki, 305–8577, Japan; 8 Department of Stem Cells and Human Disease Models, Research Center for Animal Life Science, Shiga University of Medical Science, Seta, Tsukinowa-cho, Otsu, Shiga, 520–2192, Japan; 9 PRESTO, Japan Science and Technology Agency, 4-1-8 Honcho Kawaguchi, Saitama, 332–0012, Japan; University of Kansas Medical Center, UNITED STATES

## Abstract

Pluripotency is maintained in mouse embryonic stem (ES) cells and is induced from somatic cells by the activation of appropriate transcriptional regulatory networks. *Krüppel-like factor* gene family members, such as *Klf2*, *Klf4* and *Klf5*, have important roles in maintaining the undifferentiated state of mouse ES cells as well as in cellular reprogramming, yet it is not known whether other *Klf* family members exert self-renewal and reprogramming functions when overexpressed. In this study, we examined whether overexpression of any representative *Klf* family member, such as *Klf1–Klf10*, would be sufficient for the self-renewal of mouse ES cells. We found that only *Klf2*, *Klf4*, and *Klf5* produced leukemia inhibitory factor (LIF)-independent self-renewal, although most KLF proteins, if not all, have the ability to occupy the regulatory regions of *Nanog*, a critical Klf target gene. We also examined whether overexpression of any of *Klf1-Klf10* would be sufficient to convert epiblast stem cells into a naïve pluripotent state and found that *Klf5* had such reprogramming ability, in addition to *Klf2* and *Klf4*. We also delineated the functional domains of the *Klf2* protein for LIF-independent self-renewal and reprogramming. Interestingly, we found that both the N-terminal transcriptional activation and C-terminal zinc finger domains were indispensable for this activity. Taken together, our comprehensive analysis provides new insight into the contribution of *Klf* family members to mouse ES self-renewal and cellular reprogramming.

## Introduction

Mouse embryonic stem (ES) cells are derived from the inner cell mass of the blastocyst and can be maintained indefinitely in a self-renewing state in culture [1,2]. The ability to direct the differentiation of ES cells toward a specific cell fate is a highly pursued goal in regenerative medicine [3]. However, the utilization of ES cells for therapeutic purposes will require a better understanding of the molecular mechanisms underlying the regulation of pluripotency [4,5]. Previous studies revealed that the pluripotency of ES cells is maintained by multiple soluble factors, such as LIF [6,7], and by nuclear factors [8–15], including putative core transcription factors such as *Oct3/4*, *Sox2*, *Klf* family members and *Nanog*. A reduction in extracellular-signal-regulated kinase (ERK) activity strongly promotes pluripotency. Activation of the fibroblast growth factor (Fgf)4–Fgf receptor(R)–ERK pathway destabilizes the pluripotent state and promotes a primed state [16–18]. Furthermore, the simultaneous inhibition of ERK and glycogen synthase kinase-3 (Gsk3) beta (with the inhibitor 2i) dramatically stabilizes the self-renewal process of mouse ES cells [19]. However, the precise molecular mechanisms of self-renewal remain elusive.

Induced pluripotent stem cells (iPSCs) can be derived from lineage-restricted cells, such as fibroblasts, by the forced expression of defined transcription factors [20,21]. Although previous studies have indicated that Krüppel-like transcription factors (Klfs) are essential for the reprogramming of somatic cells into a pluripotent state, the molecular mechanisms underlying these processes remain unknown [10,11,22]. The processes involved in cellular reprogramming from somatic cells and the epiblast to generate iPSCs might be similar.

Epiblast stem cells (EpiSCs) are pluripotent stem cells derived from the epiblast of embryos at the egg cylinder stage and retain the ability to differentiate into all three embryonic germ layers [23,24]. The properties and gene expression patterns of EpiSCs are very similar to those of human ES cells derived from embryos at the blastocyst stage. ES cells and EpiSCs are said to be in a naïve or in a primed state of pluripotency, respectively [25]. EpiSCs can be converted into iPSCs by reprogramming factors such as *Nanog*, *Esrrb*, and *Klf* family members including *Klf2* and *Klf4* [26,27]. The processes of self-renewal and cellular reprogramming share common transcription factors, indicating that both processes might be governed by similar molecular mechanisms.

Previous analysis indicated that expression of *Klf2*, *Klf4* and *Klf5* is associated with an undifferentiated state in mouse ES cells, and loss-of-function gene knockout (KO) studies indicated that a triple KO of *Klf2*, *Klf4* and *Klf5* resulted in defective self-renewal, and the introduction of *Klf2*, *Klf4*, or *Klf5*, but not *Klf10* rescued the defective self-renewal phenotype [11]. Our previous analysis of *Klf5* KO ES cells indicated that a lack of *Klf5* resulted in the spontaneous differentiation of mouse ES cells and that the phenotype was rescued by *Klf4* expression. Overexpression of *Klf2*, *Klf4*, or *Klf5* is sufficient to maintain the undifferentiated state of mouse ES cells in the absence of LIF [10,28,29], yet it is still unknown whether other *Klf* family members have such a function, and the functional domains required for self-renewal and cellular reprogramming are not clear.

Here we report a comprehensive analysis of which of the *Klf* family members could achieve self-renewal in mouse ES cells and found that only *Klf2*, *Klf4*, and *Klf5* possessed the ability for LIF-independent self-renewal, although most of the Klf proteins (Klf1–Klf10) could occupy the regulatory regions of *Nanog*. We also found that *Klf5*, in addition to *Klf2* and *Klf4*, had the ability to reprogram EpiSCs into a naïve pluripotent state. We also delineated the functional domains of *Klf* that could enable LIF-independent self-renewal and reprogramming. Remarkably, we found that both the N-terminal transcriptional modulation and C-terminal zinc finger DNA-binding domains were required for LIF-independent self-renewal and cellular reprogramming. This indicated that the ability to reprogram EpiSCs into naïve pluripotent stem cells is correlated with the ability to maintain the pluripotent state. Taken together, our results provide a comprehensive view on the mechanisms involved in self-renewal and reprogramming by *Klf* family members.

## Materials and Methods

### Plasmids

The coding regions of mouse *Klf1–Klf10* were amplified from cDNAs derived from mouse ES cells or tissues, sequenced, and then introduced into the multicloning site of a pPB-FLAG-HA-CAG-ireshygroR vector (Sanger Institute, Cambridge, UK).

### Culture of Mouse ES Cells and Overexpression of *Klf*

The OCRG9 ES cell line expressing green fluorescent protein (GiFP) (Oct3/4-CFP-irespuroR::Rex1-GFP) was generated as described previously [30] and a gift from Dr. Hitoshi Niwa (Kumamoto University). All ES cells were maintained in mouse ES medium consisting of Dulbecco’s modified Eagle’s medium supplemented with 10% fetal bovine serum, 1 mM non-essential amino acids, 100 μM 2-mercaptoethanol, 1 mM L-glutamine and 1,000 U/ml LIF on gelatin coated dishes as described [10]. In experiments, 1 × 10^7^ ES cells were electroporated with 15 μg of total DNA (piggyBac transposase and Klf plasmid; pPB-CAG-FLAG-HA-Klf-ireshygroR) and cultured in the presence of 150 μg/ml hygromycin B with or without LIF for 7 days; 1 μg/ml puromycin was included in the medium used for the LIF-independent self-renewal assay to select Oct3/4-positive puromycin-resistant colonies.

### Generation of EpiSCs with Stable Overexpression of Klfs

Mouse ΔPE-Oct3/4 EpiSCs were kindly provided by Dr. Austin Smith (Cambridge University) [27]. Transient transfection of mouse EpiSCs was performed on fibronectin-coated dishes seeded with 1 × 10^6^ cells using 12 μl Lipofectamine 2000 reagent (Invitrogen) with 4 μg total DNA (piggyBac transposase and Klf plasmid) per well of a six-well culture plate. The EpiSCs were cultured in N2B27 medium supplemented with human activin A (10 ng/ml; R&D) and basic fibroblast growth factor (bFgf) (5 ng/ml; Wako Pure Chemical Industries) as described previously [27] and then selected with 250 μg/ml hygromycin B and 1 μg/ml puromycin for 7 days starting from 24 h post-transfection.

For the chimera experiment, 129 EpiSCs were generated as described previously and obtained from Dr. Paul Tesar (Case Western Reserve University) [24] and transfected with a piggyBac transposase and floxed Klf5 plasmid, and cultured in the presence of 200 μg/ml G418.

### Reprogramming Assays

Wells of six-well plates were coated with fibronectin and EpiSCs were plated at a density of 2–4×10^4^ cells per well in EpiSC culture medium. After 24 h, the medium was replaced with NDiff227 medium (StemCells Inc.) supplemented with LIF (1000 U/ml) and 2i [containing a mitogen/extracellular signal-regulated kinase (Mek) inhibitor, PD0325901, 1 μM (Cayman) and the Gsk3 inhibitor CHIR99021, 3 μM (Cayman)] and changed every 24 h.

### Blastocyst Injection

Two independent iPS cell lines reprogrammed from 129 EpiSCs were transfected with a piggyBac transposase, UbC-GFP plasmid (from the Sanger Institute) and pCAG-NLS-Cre (a generous gift from Dr. Andras Nagy, Samuel Lunenfeld Research Institute). GFP-positive and Klf5 cassette-negative colonies were screened by polymerase chain reaction (PCR). The GFP-labeled iPSCs were injected into blastocysts that were transferred into surrogate mice. Embryos (9.5 and 13.5 dpc) were recovered and inspected under a fluorescence microscope.

### Western Blot Analysis

ES cells and EpiSCs were lysed on ice for 10 min in RIPA buffer (50 mM Tris-HCl pH 7.5, 150 mM NaCl, 0.5% sodium deoxycholate, 1% NP-40, 0.1% sodium dodecyl sulfate (SDS)) supplemented with a complete protease inhibitor cocktail (Roche). The extracts were clarified by centrifugation at 20,000 *g* for 5 min and suspended in sample buffer. Ten micrograms of cell extract were resolved on 10% SDS polyacrylamide gels and transferred onto polyvinylidene fluoride membranes (Millipore). Anti-hemagglutinin (HA; 1:1000) and anti-β-actin (1:4000) antibodies were used for western blotting. Proteins were detected using Immobilon kits (Millipore).

### Chromatin Immunoprecipitation (ChIP) Assay

This assay was conducted as described [31]. The cells were fixed with 1% formaldehyde, and then glycine was added to a final concentration of 0.125 M. The cells were collected in SDS lysis buffer (50 mM Tris-HCl pH 8.1, 1% SDS, 10 mM EDTA, and protease inhibitors from Nacalai Tesque). The samples were sonicated and then centrifuged at 15,800 *g* at 4°C for 15 min. After an aliquot (whole-cell extract) had been removed as an input sample, the supernatants were diluted in ChIP dilution buffer (16.7 mM Tris-HCl pH 8.1, 16.7 mM NaCl, 1.2 mM EDTA, 1.1% Triton X-100, 0.01% SDS). The diluted samples were precleared with 50 μl of protein G Sepharose beads (GE Healthcare), and then the supernatants were incubated with 4 μg of normal mouse IgG (Santa Cruz) or an anti-FLAG-M2 antibody (Sigma-Aldrich). The immunocomplexes were collected by incubation with 100 μl of protein G Sepharose beads (GE Healthcare), and then washed with the following buffers: low salt wash buffer (0.1% SDS, 1% Triton X-100, 2 mM EDTA, 150 mM NaCl, and 20 mM Tris–HCl pH 8.1); high salt wash buffer (0.1% SDS, 1% Triton X-100, 2 mM EDTA, 500 mM NaCl, and 20 mM Tris–HCl pH 8.1); and LiCl wash buffer (0.25 mM LiCl, 1% IGEPAL-C630, 1% sodium deoxycholate, 1 mM EDTA, and 10 mM Tris–HCl pH 8.1). Finally, the beads were washed twice with 1 ml of TE buffer (1 mM EDTA and 10 mM Tris–HCl pH 8.0). The immunocomplexes were then eluted by adding 200 μl of elution buffer (10 mM DTT, 1% SDS, 100 mM NaHCO_3_). After reversal of cross-linking by adding NaCl, the remaining proteins were digested with proteinase K. The purified DNA was analyzed using quantitative (q)PCR to determine which fragments were present in the precipitate. The primers for qPCR were as follows: 5′-gaggatgccccctaagctttccctccc-3′ and 5′-cctcctaccctacccaccccctattctccc-3′ for the *Nanog* promoter; and 5′-tcagcactaaccatacaagttcatc-3′ and 5′-agcgaagaggtggctggtag-3′ for the *Nanog* enhancer.

### Immunohistochemistry and Alkaline Phosphatase Assay

Cells were fixed in 4% paraformaldehyde for 10 min at 4°C and incubated overnight at 4°C with anti-Nanog antibodies. After a brief wash with phosphate-buffered saline + 0.1% Tween-20, the cells were detected in parallel by Cy3 fluorescence emissions, respectively. Nuclei were stained with Hoechst 33342 (Molecular Probes). The images were captured using a BIOREVO BZ-9000 fluorescence microscope (Keyence). Alkaline phosphatase assays to evaluate the pluripotent state were performed using leukocyte Alkaline Phosphatase kits (Sigma-Aldrich).

### Reverse-Transcription (RT) qPCR Analysis

For RT-qPCR analysis, first-strand cDNA was synthesized from total RNA using a QuantiTect Reverse Transcription kit (Qiagen). qPCR was performed with SYBR Premix Ex Taq II (Takara) and analyzed on a Thermal Cycler Dice Real Time System (TP850; Takara). The amount of target RNA was estimated using an appropriate standard curve and divided by the estimated amount of β-actin.

### Statistical Analysis

Statistical analyses were performed using unpaired Student’s *t* tests with Microsoft Office Excel. Data are expressed as the mean with standard error. Differences between means were considered significant at *P* < 0.05.

## Results

**Fig 1:**
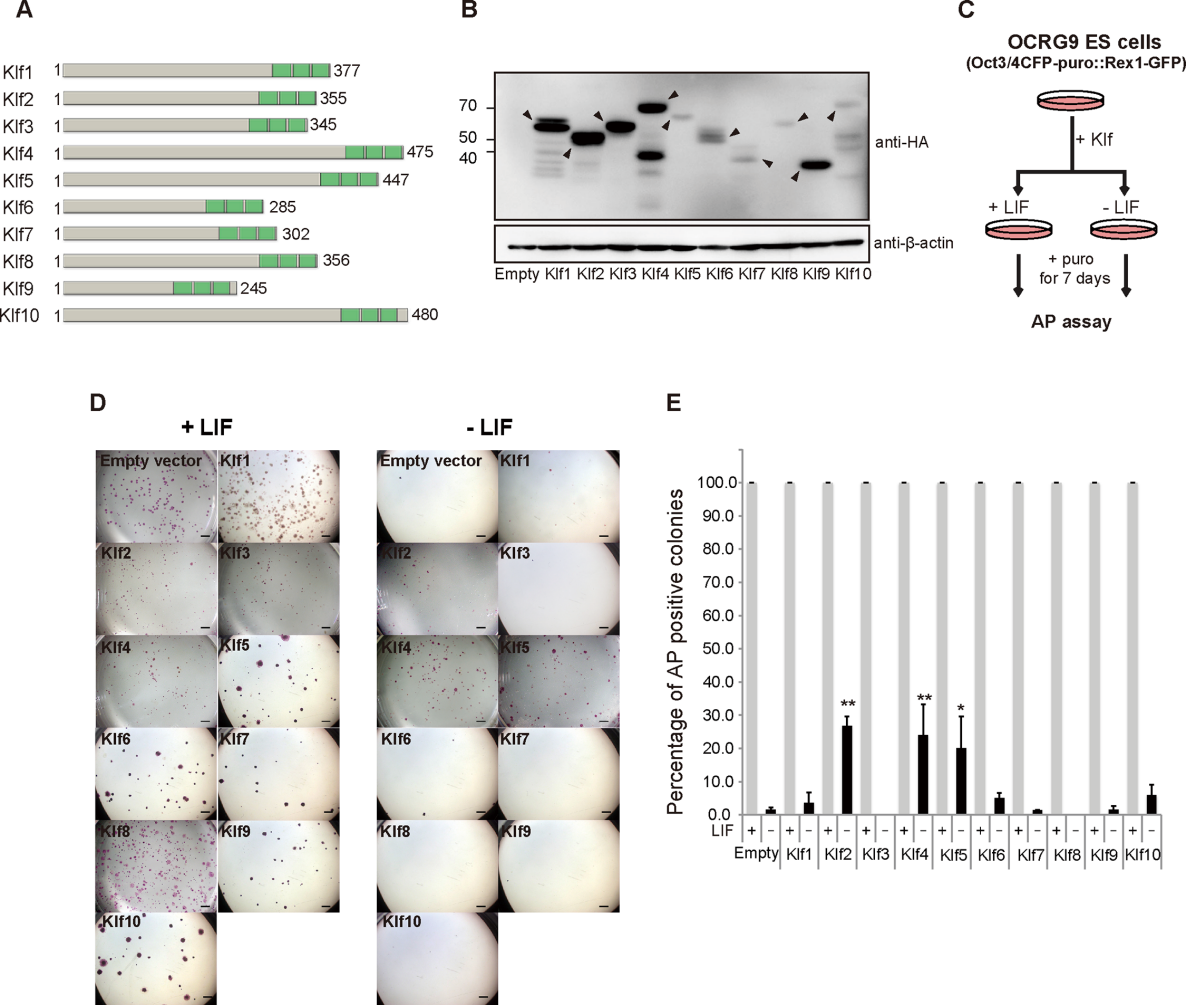
Comprehensive identification of *Krüppel-like factor* (*Klf*) family members whose overexpression achieves leukemia inhibitory factor (LIF)-independent self-renewal of mouse embryonic stem (ES) cells. (A) A schematic representation of Klfs. These proteins have highly homologous C-terminal DNA binding domains characterized by three C_2_H_2_ zinc finger motifs, shown in green boxes. The number of amino acids is shown to the left. (B) Western blot of proteins from mouse ES cells overexpressing FLAG-HA epitope-tagged Klf. Anti-HA and anti-β-actin antibodies were used to detect HA-tagged Klf and endogenous β-actin, respectively. Arrowheads indicate HA-tagged Klf proteins. (C) A schematic illustration of the experimental outline to assay the ability of a Klf protein to maintain self-renewal. (D) Generation of colonies from ES cells carrying either the empty vector or a Klf expression vector in the presence or absence of LIF. Scale bar: 2 mm. (E) The percentage of AP-positive colonies. Asterisks indicate statistical significance. *P < 0.05; **P < 0.01.

**Fig 2:**
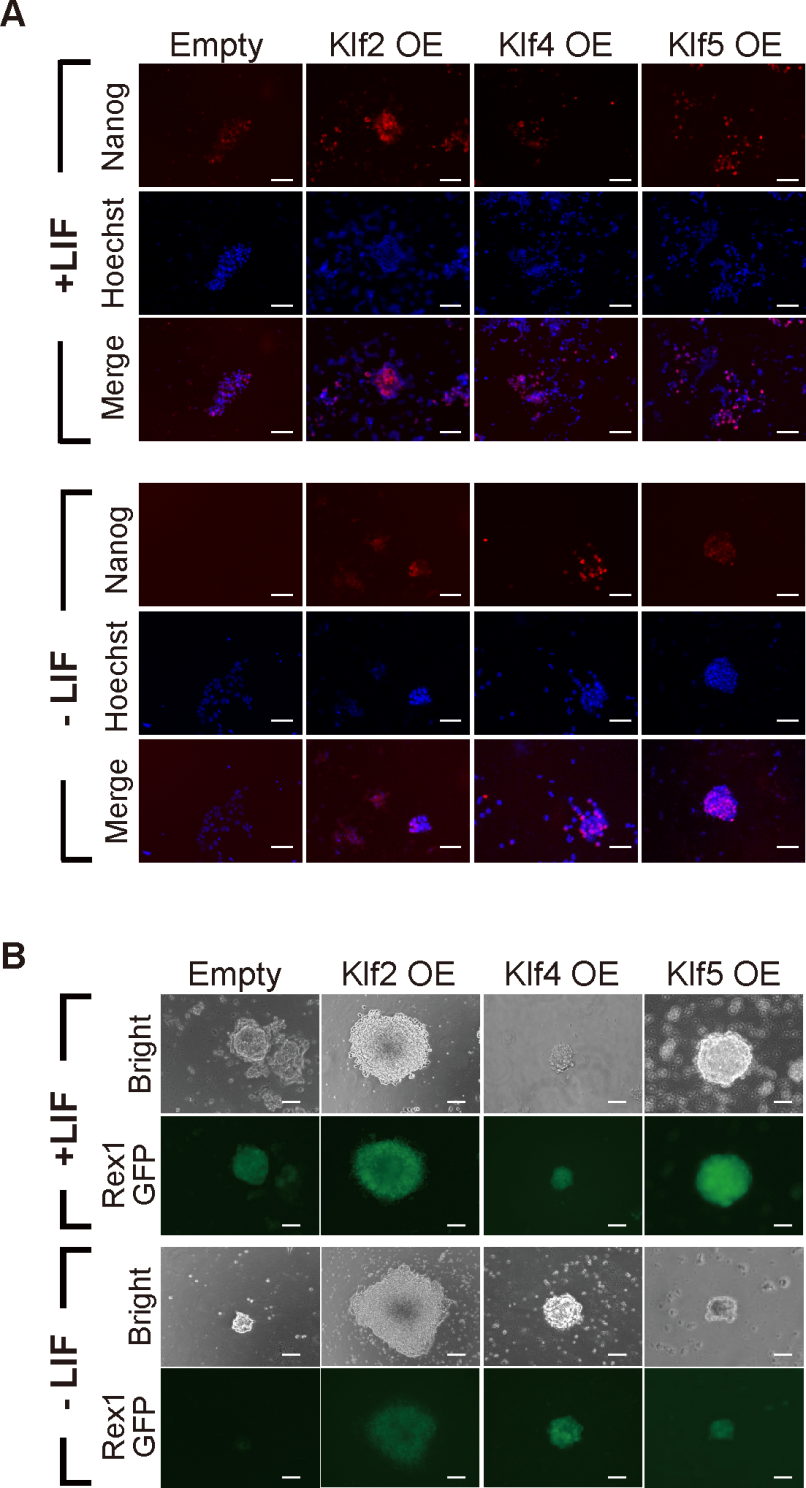
Expression of pluripotency-related markers in Klf-overexpressing mouse ES cells. (A) Immunostaining for Nanog in mouse ES cells overexpressing (OE) a Klf protein, cultured in the presence or absence of LIF. (B) Rex1-GFP expression in Klf-overexpressing ES cells grown in the presence or absence of LIF. Scale bar: 100 μm.

Although previous reports indicated that overexpression of *Klf2*, *Klf4*, or *Klf5* achieves LIF-independent self-renewal of mouse ES cells [10,11,28,29], it is still unknown whether other *Klf* family members might have similar activity. Therefore, we attempted a comprehensive investigation into whether overexpression of representative *Klf* family members would be sufficient to maintain the undifferentiated state of mouse ES cells. First, we surveyed the phylogenetic tree of the *Klf* family, which is composed of four subclasses, and selected *Klf1–Klf10* out of 18 members, because they represent the four subfamilies (data not shown) [32]. After a FLAG-HA epitope tag was introduced into the N-terminus of each *Klf* to monitor the protein expression level (Fig 1A), the expression vector was introduced into OCRG9 (Oct3/4-CFP-irespuroR:: Rex1-GFP) ES cells [30], which possesses a *Rex1-*GFP pluripotency marker. Western blot analysis confirmed that the epitope-tagged *Klf* proteins were overexpressed in the ES cells at variable levels (Fig 1B). Evaluation of relative expression levels of epitope-tagged *Klf* proteins normalized to β-actin showed that levels of overexpressed Klf6, Klf7, Klf8 and Klf10 protein were low, but not significantly different from that of Klf5 (S1 Fig). The ES cells were cultured in the presence of puromycin to select Oct3/4-positive cells, in the presence or absence of LIF (Fig 1C and 1D). All the samples, including those expressing *Klf1*–*Klf10*, showed many undifferentiated alkaline phosphatase (AP)-positive colonies in the presence of LIF, yet only cells overexpressing *Klf2*, *Klf4* or *Klf5* led to significant numbers of AP-positive colonies in the absence of LIF (Fig 1D and 1E). We performed immunohistochemistry on colonies cultured in the absence of LIF and confirmed high levels of endogenous Nanog protein (Fig 2A). We also confirmed GFP fluorescence driven by the *Rex1* promoter (Fig 2B), indicating that the undifferentiated state of pluripotency had been maintained.

**Fig 3:**
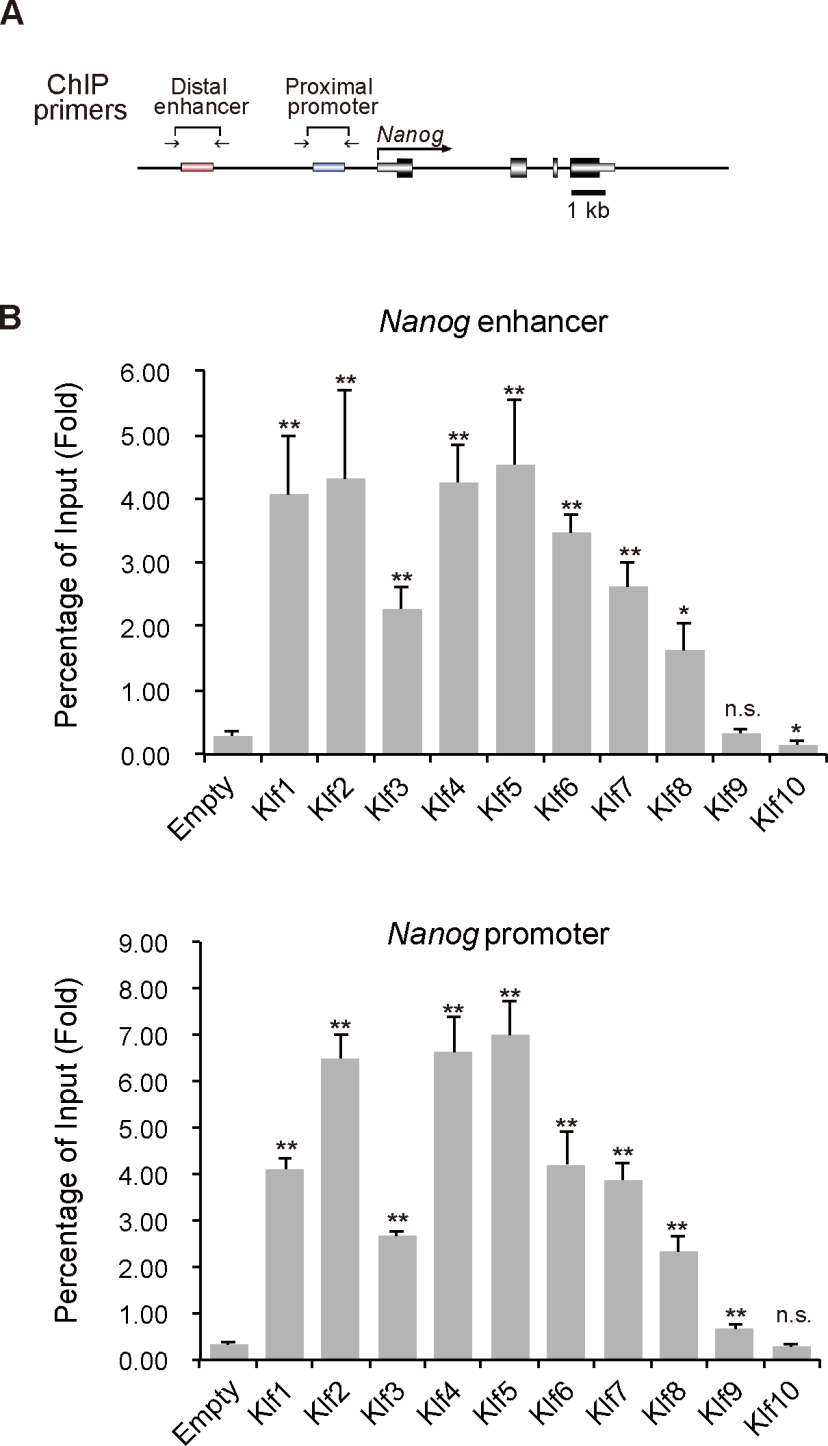
ChIP analysis of the binding of epitope-tagged Klf to *Nanog* regulatory regions in mouse ES cells. (A) Schematic representation of the *Nanog* proximal promoter and distal enhancer. Two key regulatory regions (the enhancer and proximal promoter) of *Nanog* are shown as red and blue bars, respectively. Horizontal arrows indicate the primers used for PCR in site-specific ChIP assays. (B) ChIP analysis of the binding of Klf to the promoter and enhancer of *Nanog*. Asterisks indicate statistical significance. *P < 0.05; **P < 0.01; n.s.; not significant.

Overexpression experiments clearly indicated that *Klf2*, *Klf4* and *Klf5*, but not any other *Klf* family member, were able to maintain the undifferentiated state in the absence of LIF. It is not known why only these three members have this ability. To investigate the molecular mechanism underlying the pluripotency maintained by *Klf* family members, we focused on the *Nanog* locus, because this gene is important for the self-renewal of mouse ES cells [9,14,33] and is controlled by the Klf2, Klf4, and Klf5 transcription factors [10,11]. Previous reports indicated that Klf2, Klf4 and Klf5 activate *Nanog* through binding to the promoter and its 3′ enhancer [11]. When the overexpressed Klf proteins were examined for binding affinities to the promoter and enhancer (Fig 3A), most of them except for Klf9 and Klf10 showed efficient recruitment to the regulatory regions at similar levels to Klf2, Klf4 and Klf5 (Fig 3B). Currently, it is not clear why Klf1, Klf3, Klf6, Klf7, and Klf8 are not sufficient to maintain self-renewal of mouse ES cells, although they have the ability to bind to *Nanog* regulatory regions.

**Fig 4:**
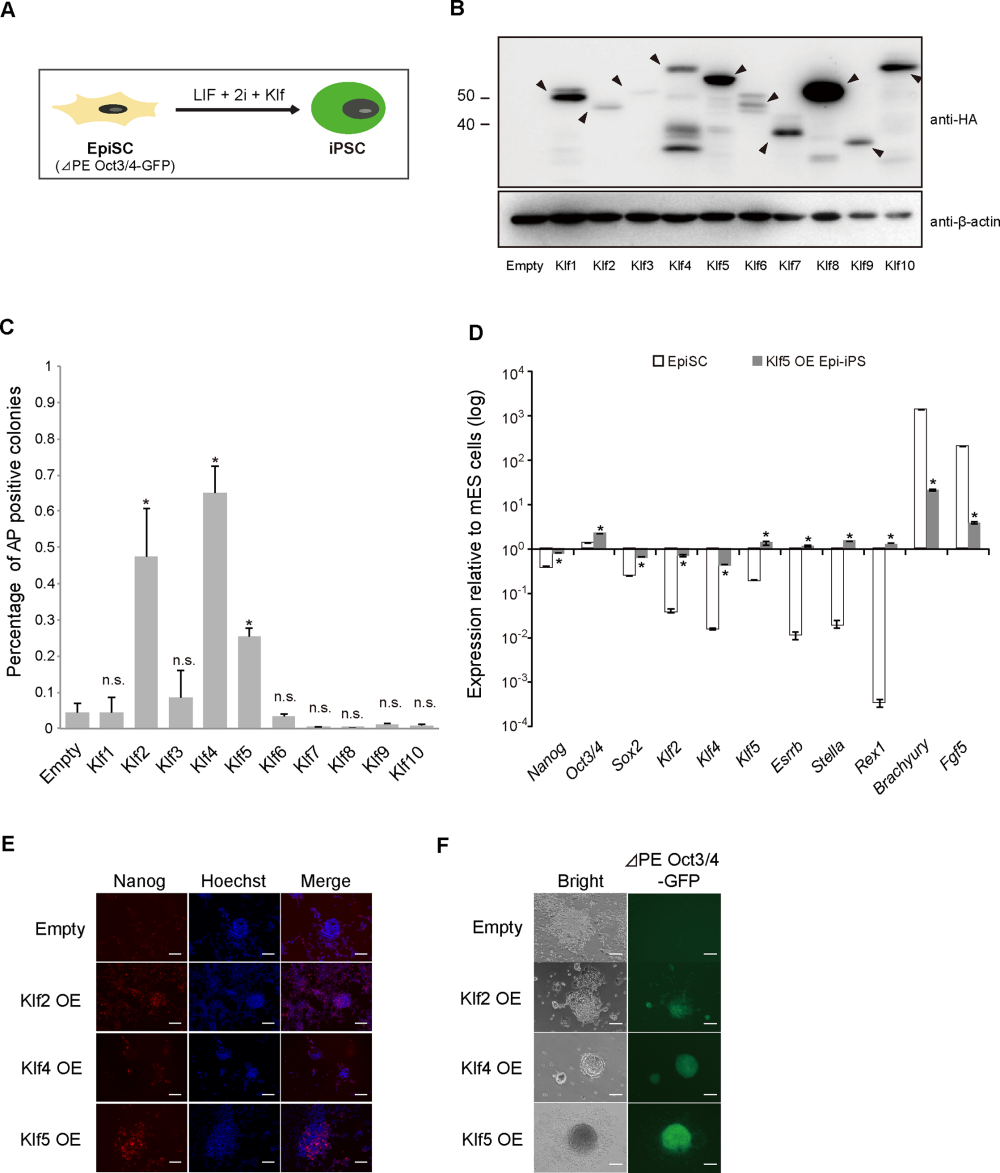
Identification of the ability of Klf family members to reprogram EpiSCs into iPSCs. (A) Experimental outline for evaluating the reprogramming activity of a Klf protein. EpiSCs carrying Oct3/4-∆PE-GFP were cultured in N2B27 medium supplemented with LIF, the Mek inhibitor PD0325901, and the Gsk3 inhibitor CHIRON (CHIR99021) for 5–7 days and were then subjected to AP assays. (B) Western blot analysis of EpiSCs expressing epitope-tagged Klf. (C) Percentage of AP-positive colonies generated from EpiSCs overexpressing a Klf protein. (D) RT-qPCR analysis of iPSCs. (E) Immunostaining for Nanog in iPSCs generated by overexpression of Klf2, Klf4 and Klf5. (F) GFP fluorescence in EpiSCs generated by Klf2, Klf4 and Klf5 overexpression. Scale bar: 100 μm. Asterisks indicate statistical significance. *P < 0.05; **P < 0.01; n.s.; not significant.

**Fig 5:**
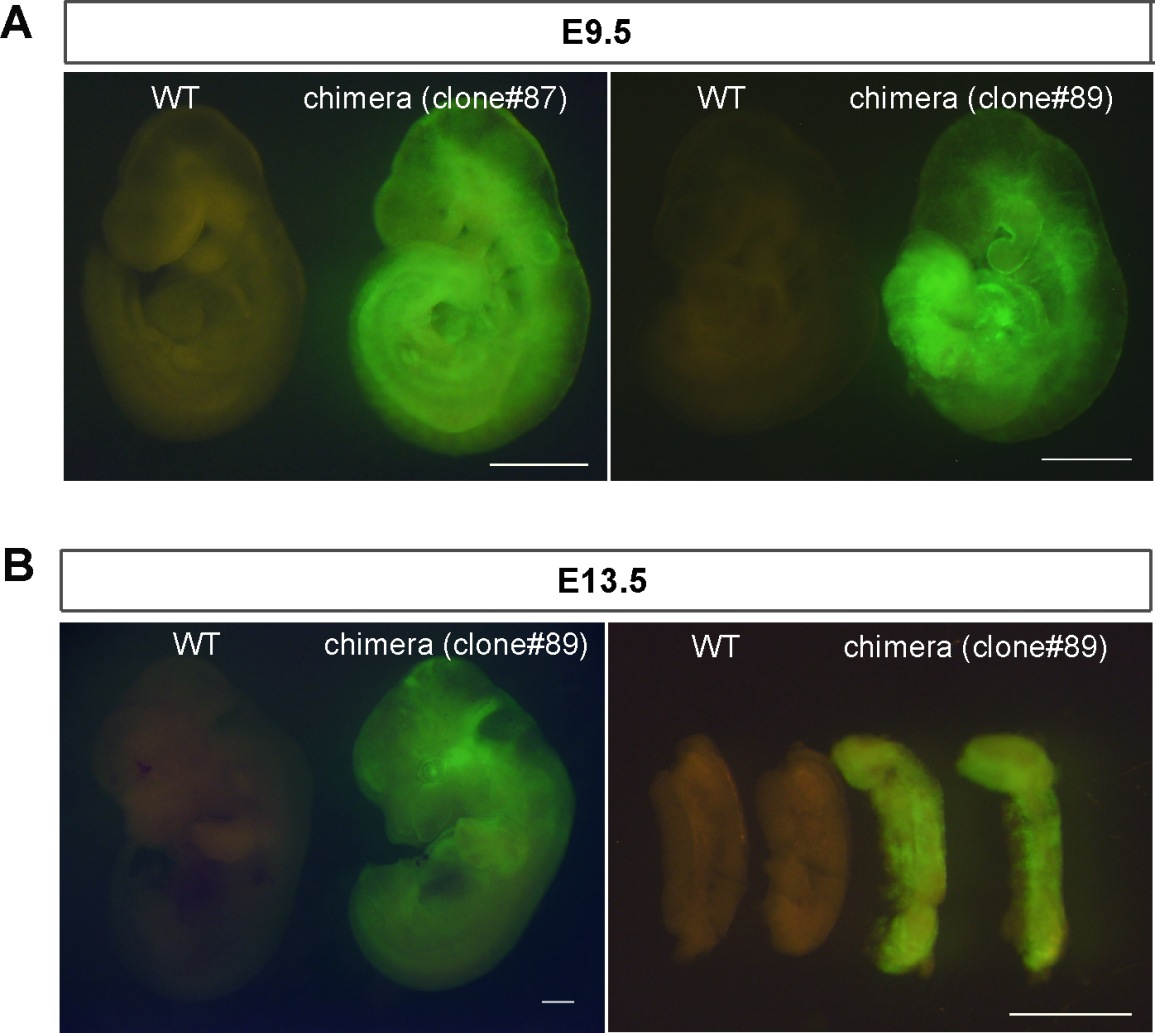
Chimera formation with iPSCs reprogrammed from EpiSCs. (A) Chimeras at E9.5 generated from two independent iPSC lines (#87 and 89). (B) Chimera and its genital ridge at E13.5 generated from iPSC line #89. Scale bar: 1 mm.

Mouse EpiSCs can spontaneously reprogram into the naïve state of iPSCs, but at a very low rate [34]. Transient expression of somatic reprogramming factors, such as *Klf4*, *Nanog*, *Esrrb*, and *Klf2*, cause more efficient and quicker reversion of EpiSCs into iPSCs, implying that this reversion mimics cellular reprogramming from fibroblasts into iPSCs. Previous analysis also indicated that *Klf2* and *Klf4*, but not *Klf5*, enhance reprogramming of EpiSCs into iPSCs [28]. We comprehensively examined the reprogramming ability of Klfs (Fig 4A). Expression vectors carrying epitope-tagged *Klf* genes were introduced into EpiSCs and protein levels were confirmed using an anti-HA antibody (Fig 4B). When cells were transferred from EpiSC cultures containing bFGF and activin A into ES medium containing LIF and 2i, only the EpiSCs overexpressing *Klf2*, *Klf4*, or *Klf5* generated significant numbers of AP-positive colonies (Fig 4C). A previous report indicated that *Klf5* expression could not enhance reprogramming of EpiSCs to iPSCs [28]; therefore, we carefully evaluated whether iPSCs generated by *Klf5* overexpression were indeed pluripotent. The RT-qPCR analysis showed elevated expression levels of naïve markers, such as *Nanog*, *Klf2*, *Klf4*, *Esrrb*, *Stella*, and *Rex1*, while the expression levels of primed state markers, such as *Fgf5* and *Brachyury* were reduced (Fig 4D). Endogenous Nanog protein was also present in the iPSCs (Fig 4E). The *Oct3/4* gene contains distal and proximal enhancers; the distal one drives naïve pluripotent stem cells, while both enhancers are required for generating primed pluripotent stem cells [27]. The iPSCs generated by transient *Klf5* expression showed strong GFP fluorescence without the presence of the proximal enhancer (Fig 4F), also indicating that the iPSCs were indeed naïve pluripotent stem cells. We then performed chimera formation, which is the gold standard assay for pluripotency (Fig 5). The iPSCs reprogrammed by overexpression of *Klf5* were injected into blastocysts and the resultant blastocysts were returned into the uterus of a surrogate mother. E9.5 fetuses generated from two independent iPSCs exhibited strong contribution of GFP-positive iPSCs (Fig 5A). Furthermore, E13.5 fetuses exhibited strong contribution of GFP-positive iPSCs throughout the whole embryo including the genital ridge (Fig 5B), indicating that iPSCs have the ability to differentiate into all three germ layers, including the germ cell lineage.

**Fig 6:**
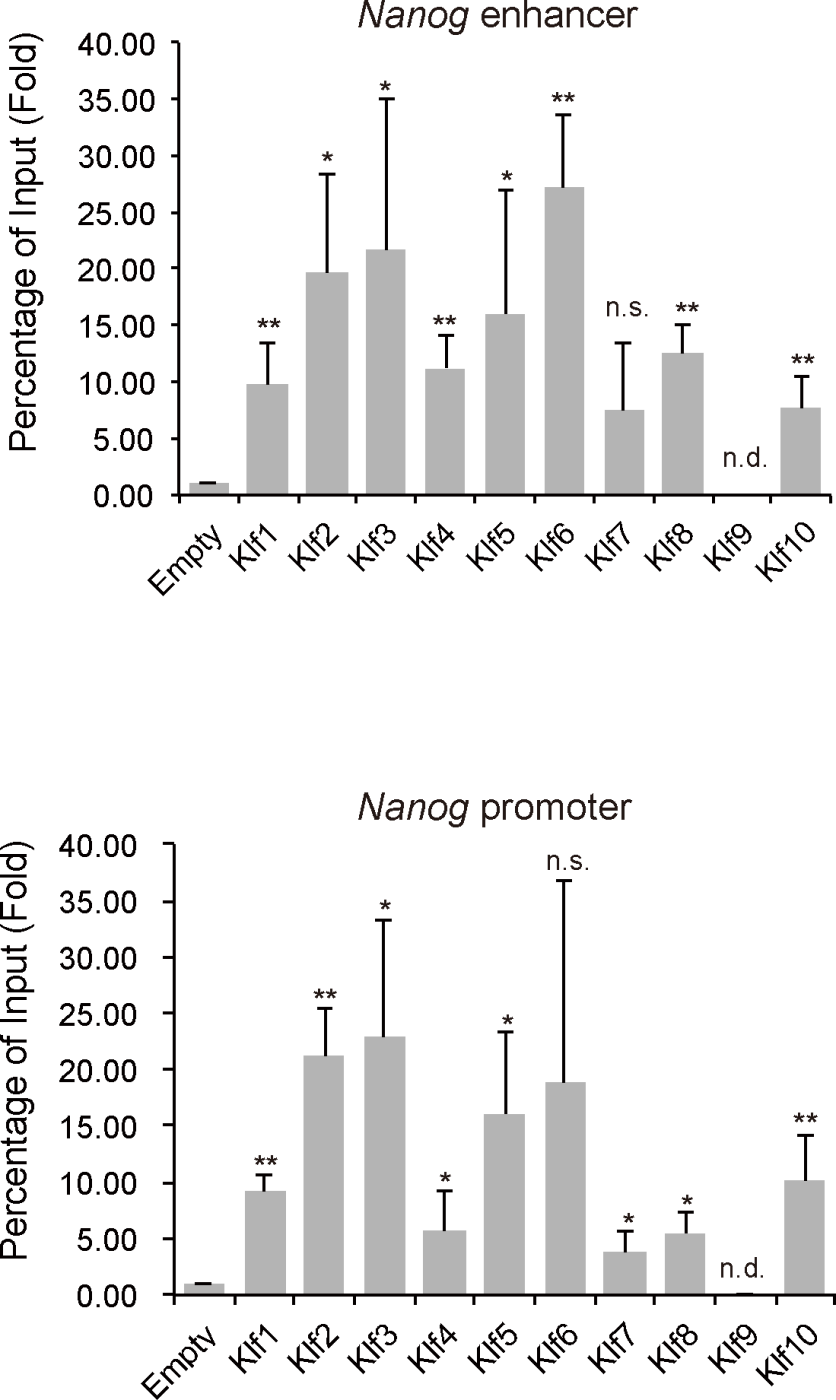
ChIP analysis of the binding of Klf to *Nanog* regulatory regions in mouse EpiSCs. ChIP experiments were performed with an anti-FLAG antibody to identify the promoter and enhancer of *Nanog*. Asterisks indicate statistical significance. *P < 0.05; **P < 0.01; n.s.; not significant; n.d.; not detected.

To gain insight into how *Klf2*, *Klf4* and *Klf5* might regulate reprogramming of EpiSCs to iPSCs, we performed ChIP assays to assess the occupation of Klf factors at the *Nanog* locus under EpiSC culture conditions. Most of the Klf proteins examined, except for Klf9, occupied the *Nanog* promoter and enhancer, indicating that occupation itself was not sufficient for reprogramming (Fig 6). Our analysis indicated that Klf2, Klf4, and Klf5 did not affect *Nanog* transcription under EpiSC culture conditions (data not shown).

**Fig 7:**
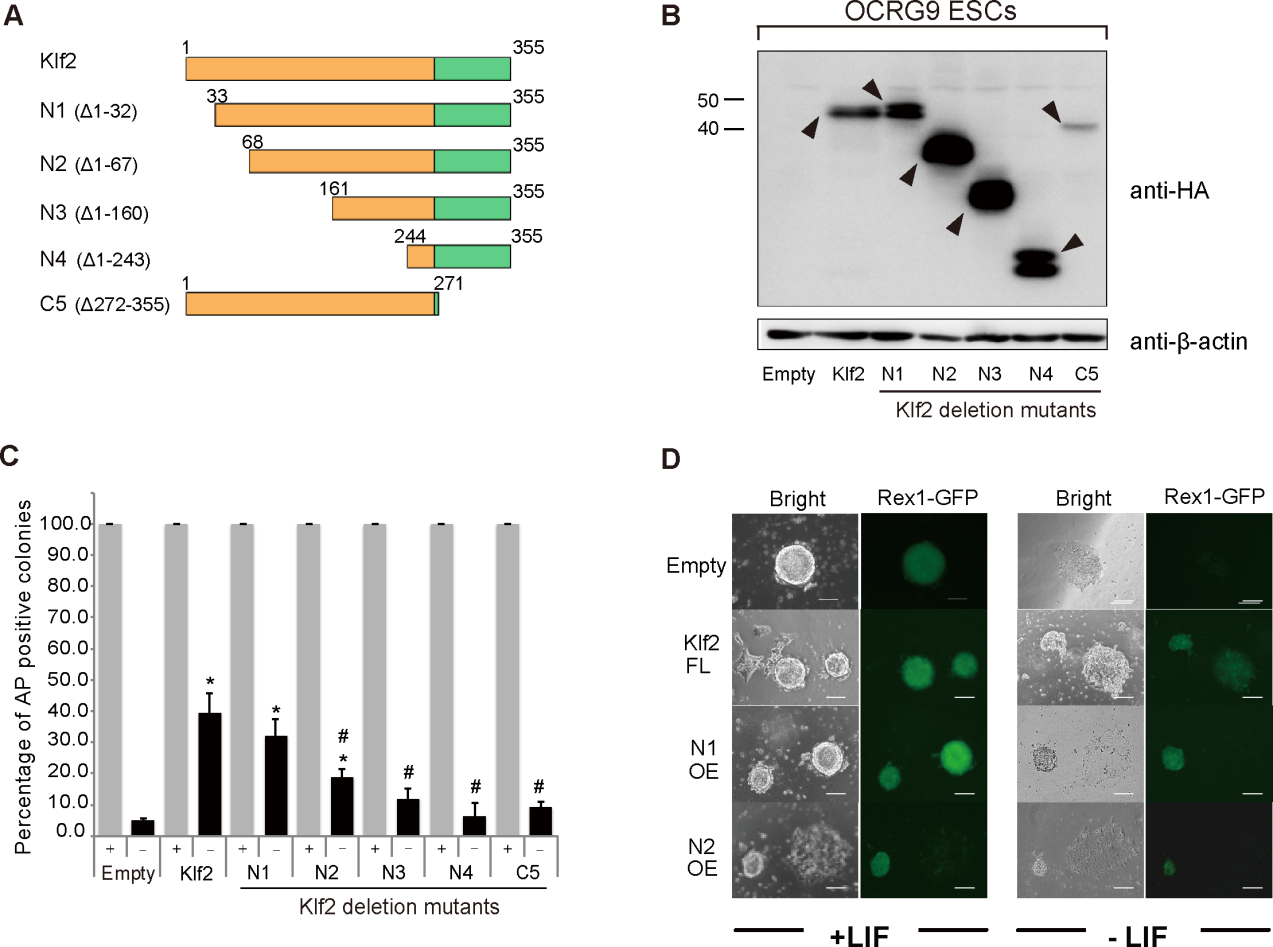
Delineation of the domains of Klf2 required for the self-renewal of mouse ES cells. (A) Schematic illustration of Klf2 deletion (Δ) mutants. Klf2 is highly homologous to Klf4 and belongs to the same subclass. A FLAG-HA tandem epitope tag was fused to the N-terminus of each of the deletion mutants. The zinc finger domain is shown as green boxes. (B) Western blot analyses of Klf2 and Klf2 deletion mutants with an anti-HA antibody. Arrowheads indicate HA-Klf proteins. (C) Percentage of AP-positive colonies in ES cells overexpressing full-length Klf2 or Klf2 deletion mutants, grown in the presence or absence of LIF. (D) Expression of the pluripotency-related marker Rex1. Rex1-GFP expression is seen in full-length Klf2, and N-terminal deletion mutants (N1, N2) in the absence of LIF. Scale bar: 100 μm. Asterisks and hash symbols indicate statistical significance. *P < 0.05 vs. empty vector; # *P* < 0.05 vs. full-length Klf; * *P* < 0.01; n.s.; not significant; n.d.; not detected.

**Fig 8:**
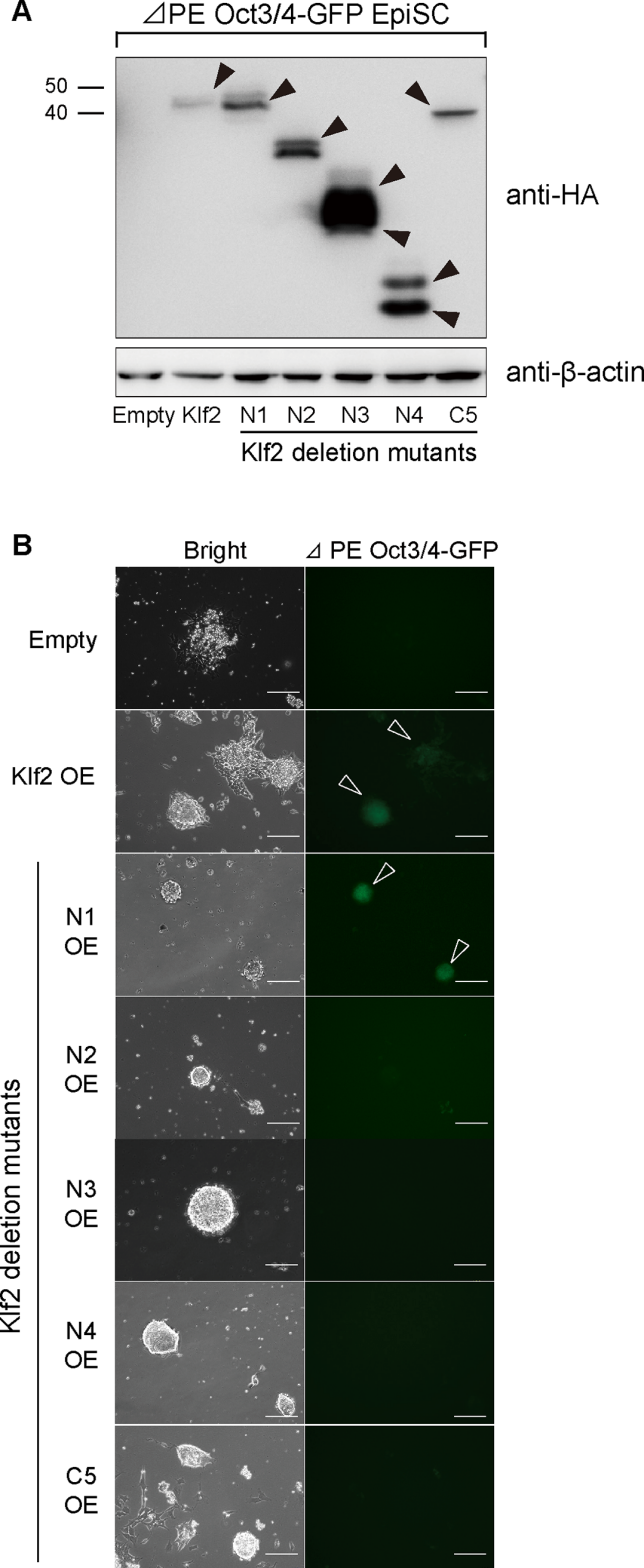
Delineation of Klf2 domains required for reprogramming EpiSCs to iPSCs. (A) Western blot analysis of EpiSCs expressing epitope-tagged Klf2 and deletion (Δ) mutants. Closed arrowheads indicate epitope-tagged Klf2 proteins. (B) Fluorescence images of a reprogrammed Oct3/4-ΔPE-GFP EpiSC colony with a Klf2 deletion domain on day 7. Open arrowheads indicate GFP fluorescence. OE, overexpression. Scale bar: 100 μm.

To delineate the functional domains required for LIF-independent self-renewal, we compared the amino acid sequences of Klf2, Klf4 and Klf5, and found that these three Klf proteins share homologous regions in the N-terminal half, which acts in transcription, as well as in the C-terminal zinc finger DNA-binding domain (S2 Fig). Because Klf2 is the smallest of these proteins, deletion mutants for *Klf2* were created (Fig 7A). Western blot analysis confirmed that exogenous Klfs were overexpressed in ES cells in the presence of LIF (Fig 7B). When LIF-independent self-renewal activity was evaluated, N-terminal deletion resulted in a reduction in AP-positive clones (Fig 7C). Deletion of two zinc fingers also abolished the efficiency of this self-renewal activity. Thus, our analysis clearly indicates that both the N-terminal transcriptional modulation domain and the C-terminal zinc finger DNA-binding domain are required for the full activity of Klfs (Fig 7). We also delineated the functional domains required for reprogramming of EpiSCs into iPSCs (Fig 8). When various *Klf* deletion mutants were overexpressed in EpiSCs in the presence of bFGF and activin A (Fig 8A), we found that both the N-terminal transcription modulation domain and the C-terminal zinc finger DNA-binding domain were required for full activity (Fig 8B). This was similar to the domains required for self-renewal.

## Discussion

Our analysis identified that only Klf2, Klf4, and Klf5 have the abilities to both maintain the undifferentiated state of mouse ES cells and to reprogram EpiSCs into iPSCs when overexpressed. We also delineated functional domains of Klf for LIF-independent self-renewal and reprogramming and found that both the N-terminal transcription modulation domain and the C-terminal zinc finger DNA-binding domain were required for LIF-independent self-renewal and reprogramming. This finding indicates that the ability to reprogram EpiSCs into naïve pluripotent stem cells is correlated with the ability to maintain the pluripotent state.

A previous report by Wang et al. [35] indicated that the reprogramming ability of Oct3/4, Nanog and Sox2 could be boosted by using a chimeric protein including VP16, a potent transcriptional activator, in place of the inherent transcriptional modulation domain, indicating that transcriptional activation is important for the reprogramming ability of those core factors. On the other hand, only a Klf4-VP16 chimeric protein provided a similar level of reprogramming activity, suggesting that the transcriptional modulation domain exerts inherent activity.

EpiSCs can be converted into iPSCs by naïve-state transcription factors such as *Nanog*, *Esrrb*, *Klf2* and *Klf4* [26, 27]. Overexpression of *Klf2*, *Klf4* or *Klf5* maintain the undifferentiated state of mouse ES cells in the absence of LIF [10, 28, 29]. It is of note that induced expression of Klf4 and Klf5 are associated with the naïve state of rabbit iPS and ES cells [36], indicating a potential conserved role of Klf factors in the naïve state among species.

Previous “omics” studies using mass spectroscopy indicated that Oct3/4 exists in a large complex containing Sall4, Tcfcp2l1, Dax1, Esrrb, and other epigenetic modifiers in mouse ES cells [37]. Similarly, Nanog binds to Sox2, Mta2, Sall4, and PolII [38]. Interestingly, Klf5 is a protein that forms a complex with Oct3/4 and Nanog [37, 38]. Our previous study [10] indicated that Klf5 is in a complex with Metastasis-associated protein (MTA2), a component of Nucleosome Remodeling Deacetylase (NuRD). Our results here will be useful for the further exploration of functional domains that contribute to self-renewal and reprogramming. It will be interesting to identify possible cofactors that are important for self-renewal and reprogramming, leading to elucidation of the molecular mechanisms involved.

## Supporting Information

S1 Fig Relative expression levels of epitope-tagged Klf protein normalized to β-actin. Signal intensities of epitope-tagged Klf protein were normalized to that of β-actin. Three independent sets of data were used to calculate statistical relevance.
(TIF)

S2 Fig Multiple sequence alignment of mouse Klf protein using ClustalW. Klf2, Klf4 and Klf5 share highly homologous C-terminal DNA binding domains characterized by three C2H2 zinc finger motifs. An * (asterisk) indicates positions with a single, fully conserved residue. A: (colon) indicates conservation between groups of strongly similar properties, scoring > 0.5 in the Gonnet PAM 250 matrix. A. (period) indicates conservation between groups of weakly similar properties scoring ≤ 0.5 in the Gonnet PAM 250 matrix.
(TIF)

S1 Table Primers used for RT-qPCR analysis. (PDF)
