# Supplementary figures and images for: Comprehensive Identification of Krüppel-Like Factor Family Members Contributing to the Self-Renewal of Mouse Embryonic Stem Cells and Cellular Reprogramming
Source: PLoS One. 2016 Mar 4;11(3):e0150715. doi: 10.1371/journal.pone.0150715 (PMC4778944; doi:10.1371/journal.pone.0150715)

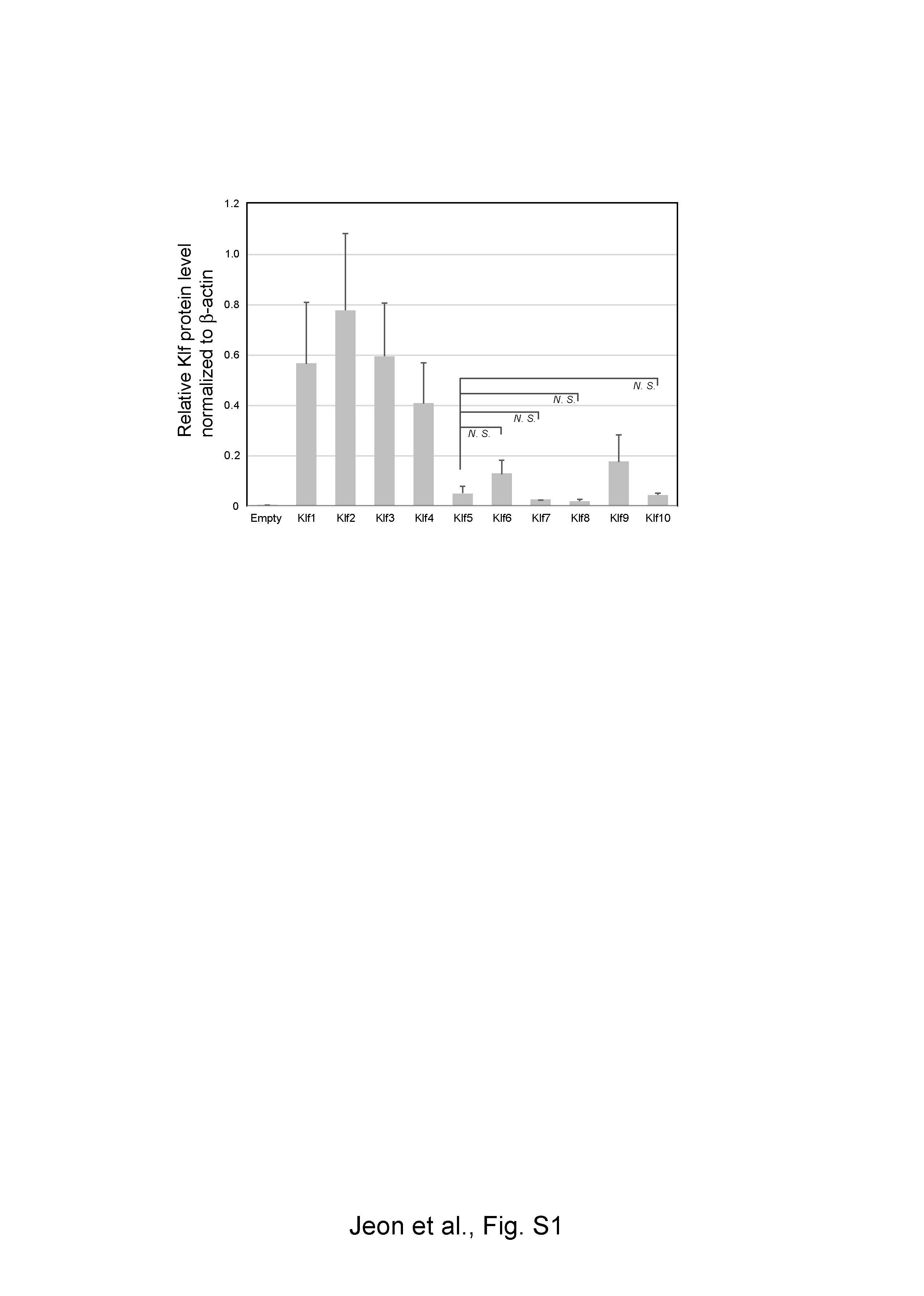

Supplement: S1 Fig — Signal intensities of epitope-tagged Klf protein were normalized to that of β-actin. Three independent sets of data were used to calculate statistical relevance. (TIF) [file pone.0150715.s001.tif]

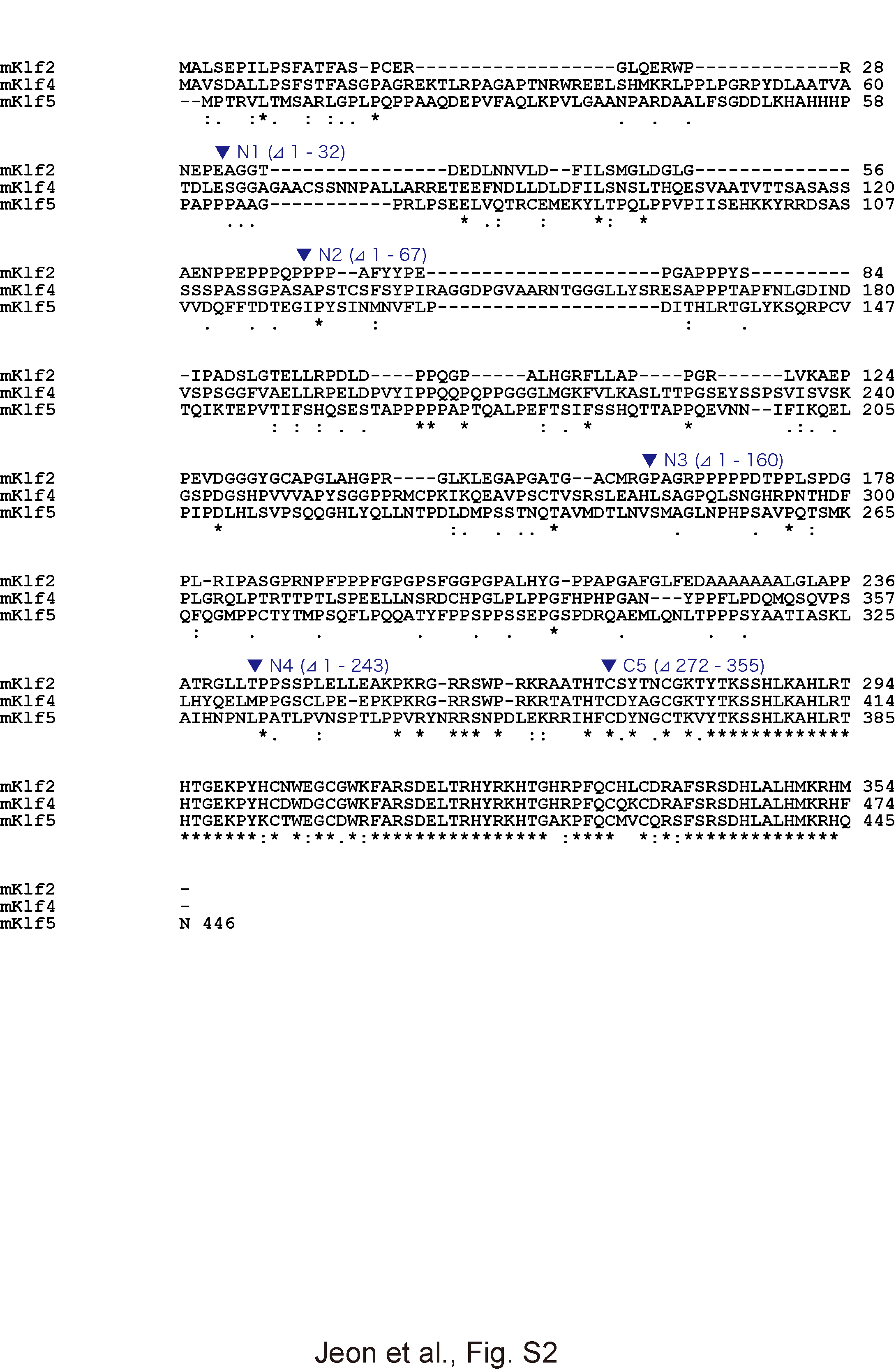

Supplement: S2 Fig — Klf2, Klf4 and Klf5 share highly homologous C-terminal DNA binding domains characterized by three C2H2 zinc finger motifs. An * (asterisk) indicates positions with a single, fully conserved residue. A: (colon) indicates conservation between groups of strongly similar properties, scoring > 0.5 in the Gonnet PAM 250 matrix. A. (period) indicates conservation between groups of weakly similar properties scoring ≤ 0.5 in the Gonnet PAM 250 matrix. (TIF) [file pone.0150715.s002.tif]
