# Supplementary material for: Comprehensive Identification of Krüppel-Like Factor Family Members Contributing to the Self-Renewal of Mouse Embryonic Stem Cells and Cellular Reprogramming
Source: PLoS One. 2016 Mar 4;11(3):e0150715. doi: 10.1371/journal.pone.0150715 (PMC4778944; doi:10.1371/journal.pone.0150715)
Supplement: S1 Table — (PDF) [file pone.0150715.s003.pdf]

Table S1: List of qRT-PCR primer sequences

Gene	Primer Sequence (Forward)	Primer Sequence (Reverse)
Oct3/4	TATTGAGTATTCCCAACGAGAAGAG	CTCAGGAAAAGGGACTGAGTAGAGT
Nanog	CTTTCACCTATTAAGGTGCTTGC	TGGCATCGGTTCATCATGGTAC
Sox2	CATGAGAGCAAGTACTGGCAAG	CCAACGATATCAACCTGCATGG
Klf2	TCGAGGCTAGATGCCTTGTGA	AAACGAAGCAGGCGGCAGA
Klf4	TACCCCTACACTGAGTCCCG	GGAAAGGAGGGTAGTTGGGC
Klf5	CGATTCACAACCCAAATTTACC	GTATGAGTCCTCAGGTGAGCTTTTA
Esrrb	ATGAATGTGAGATCACCAAACG	GTTCAGGTAGGGGCTGTTCTC
Fgf5	ACCCACTTCCTACCCAGGTT	AGTTGTTTCCCACAAGGCCA
T (Brachyury)	CATGTACTCTTTCTTGCTGGACTTC	GAGCTTGTTGGTGAGTTTGACTTT
Stella	AGGCTCGAAGGAAATGAGTTTG	TCCTAATTCTTCCCGATTTTCG
Rex1	TCCATGGCATAGTTCCAACAG	TAACTGATTTTCTGCCGTATGC
β-actin	CTGAGCGCAAGTACTCTGTGTG	GTGTAAAACGCAGCTCAGTAACAGT
